# How to Overcome Anabolic Resistance in Dialysis-Treated Patients?

**DOI:** 10.3389/fnut.2021.701386

**Published:** 2021-08-12

**Authors:** Giacomo Garibotto, Michela Saio, Francesca Aimasso, Elisa Russo, Daniela Picciotto, Francesca Viazzi, Daniela Verzola, Alessandro Laudon, Pasquale Esposito, Giuliano Brunori

**Affiliations:** ^1^Department of Internal Medicine, University of Genoa, Genova, Italy; ^2^Clinical Nutrition Unit, Istituto di Ricerca a Carattere Scientifico Ospedale Policlinico San Martino, Genova, Italy; ^3^Clinica Nefrologica, Dialisi e Trapianto, IRCCS Ospedale Policlinico San Martino, Genova, Italy; ^4^Division of Nephrology and Dialysis, Ospedale Santa Chiara, Trento, Italy

**Keywords:** nutrition, muscle, protein synthesis, hemodialysis, peritoneal dialysis, CKD

## Abstract

A current hypothesis is that dialysis-treated patients are “anabolic resistant” i. e., their muscle protein synthesis (MPS) response to anabolic stimuli is blunted, an effect which leads to muscle wasting and poor physical performance in aging and in several chronic diseases. The importance of maintaining muscle mass and MPS is often neglected in dialysis-treated patients; better than to describe mechanisms leading to energy-protein wasting, the aim of this narrative review is to suggest possible strategies to overcome anabolic resistance in this patient's category. Food intake, in particular dietary protein, and physical activity, are the two major anabolic stimuli. Unfortunately, dialysis patients are often aged and have a sedentary behavior, all conditions which *per se* may induce a state of “anabolic resistance.” In addition, patients on dialysis are exposed to amino acid or protein deprivation during the dialysis sessions. Unfortunately, the optimal amount and formula of protein/amino acid composition in supplements to maximixe MPS is still unknown in dialysis patients. In young healthy subjects, 20 g whey protein maximally stimulate MPS. However, recent observations suggest that dialysis patients need greater amounts of proteins than healthy subjects to maximally stimulate MPS. Since unneccesary amounts of amino acids could stimulate ureagenesis, toxins and acid production, it is urgent to obtain information on the optimal dose of proteins or amino acids/ketoacids to maximize MPS in this patients' population. In the meantime, the issue of maintaining muscle mass and function in dialysis-treated CKD patients needs not to be overlooked by the kidney community.

## Introduction

Dialysis-treated patients with end-stage renal disease (ESRD) have a high prevalence of protein-energy wasting (PEW), a condition of muscle and visceral protein stores loss which is not completely accounted for by a low nutrient intake (1). There is a strong association between surrogates of muscle mass and survival in this patients' population (2, 3); therefore, the need for increased medical attention for nutrition in advanced kidney disease is a high clinical priority.

**Figure 1:**
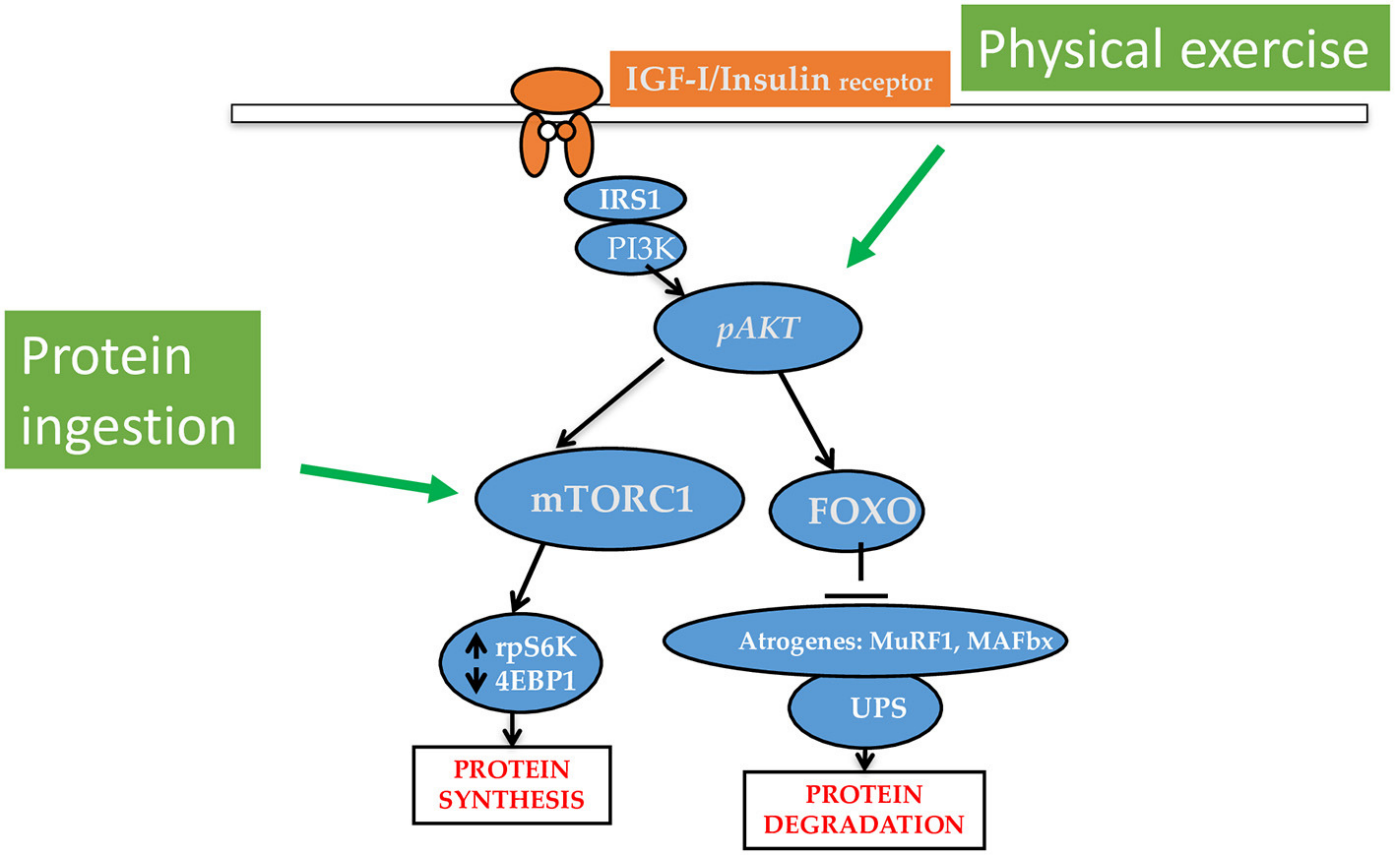
Major intracellular pathways underlying the anabolic effects of protein/amino acid/keto acid intake and physical exercise. The ingestion of protein, amino acids or keto acid of essential amino acids increases muscle protein synthesis (MPS) rates. Physical exercise upregulates phosphorylated Akt to increase MPS and decrease protein degradation. Protein ingestion and physical exercise have synergistic interaction on the stimulation of MPS.

Muscle protein synthesis (MPS), serving either the maintenance of muscle protein during fasting and body anabolism during feeding, is central to the processes of sustaining and shaping body cell mass. Dietary proteins or amino acids (AA), and physical activity are the two major anabolic stimuli that increase MPS (4). If combined, these two factors offer the major available anabolic stimulus in humans (Figure 1). Several other factors, including the dietary protein amount and composition, and the timing of protein ingestion can also influence the anabolic responses.

Recent understanding of the mechanisms which regulate protein metabolism in chronic kidney disease (CKD) has allowed us to understand the role of acidosis, insulin resistance and inflammation on intracellular signals which promote PEW by activating muscle protein degradation (MPD) (5). Even more importantly, Zhang et al. (6) were able to demonstrate that CKD stimulates chromatin-modifying, nucleolar protein 66 (NO66), that downregulates MPS via a demethylase mechanism, giving us a new perspective on the pathophysiology of uremia-related wasting syndrome. Better than to describe mechanisms leading to muscle wasting in dialysis patients, the scope of this narrative review is to analyze current understanding of the dietary and non-dietary modulators of MPS as major determinants of muscle mass in humans, in order to suggest to suggest possible strategies to stimulate MPS and, at least in part, overcome anabolic resistance in dialysis-treated patients with CKD.

## The Response of MPS to Protein Feeding

The MPS response to protein is influenced by both the amount and the type of protein, and the time of feeding.

### Protein Amount

The hyperaminoacidemia reached after protein ingestion is “primary anabolic” since it increases MPS rates, and to a minor degree, decreases muscle protein degradation (MPD) (7, 8). Several investigators have studied the optimal amount of protein for the maximal stimulation of MPS in humans (7–14). As demonstrated in early studies (14), the muscle anabolic response to AA/protein ingestion is time-limited in the rested state; after 2–3 h from AA ingestion, MPS undergoes a state of “tachiphylaxis” in spite of ongoing EAA availability and upregulation of mechanistic target of rapamycin complex 1 (mTORC1) signaling (14). This 'muscle full' condition is refractory to the intake of additional nutrient signals/substrates regulating MPS, is not induced by muscle blood flow and does not appear to be dependent on the amount of protein ingested (4, 14). Instead, the 'muscle full' set-point can be delayed by physical exercise to allow additional use of EAA for MPS (4).

The dose-response of myofibrillar MPS to increasing amounts of whey protein at rest and after exercise has been studied in young subjects by Witard et al. (8). They observed that a 20-g dose of whey protein (containing ~10 g essential AAs) is sufficient for the maximal stimulation of MPS both at rest and during physical exercise, while a greater dose stimulates amino acid oxidation. However, in elderly subjects, more protein with respect to younger subjects is needed to maximally stimulate MPS in exercised and rested muscle (15).

### Protein Type

Also AA composition of the dietary protein influences the MPS response. Lower essential AA (EAA) content, particularly of leucine, lysine, and/or methionine has been shown to be responsible for the lower anabolic capacity of low biological vs. high biological value proteins (16, 17). Apart from their AA content, the nutritional value of proteins ingested with the diet depends also on a series of factors, including their chemical score, net utilization, and digestibility corrected AA score (PDCAAS) (18). Many of these factors are better for animal-based than for plant-based protein sources (19). Both the PDCAAS and the Digestible Indispensable AA Score (DIAAS) are used to assess the ability of dietary protein to satisfy the body's AA requirements (18, 19).

Protein ingestion and physical exercise possess a synergistic interaction on the stimulation of MPS (20, 21). Protein ingestion provides, among the EAA and non-essential AA needed for MPS, also leucine, which stimulates protein translation. In addition, protein and carbohydrate ingestion increase serum levels of insulin, which has a mild stimulatory effect on MPS (20–22) and decreases MPD. Another factor which makes protein differently anabolic is the splanchnic AA removal from circulation (23, 24). As an example, proteins from soy undergo an important splanchnic first-pass, whereas milk proteins are directed to peripheral sites. In comparison to soy or carbohydrate, milk consumption has a greater anabolic effect in young subjects in the post-exercise period (25).

The content of essential AA in plant vs. animal-based proteins is likely the major determinant of their different muscle anabolic effects. Plant proteins have lower essential AA content, in particular of methionine and lysine, than animal-based and muscle protein (17). Of note, the intake of AAs is highest in diets containing prevalently meat protein, followed by fish, and is lowest in diets containing only plant protein (26); accordingly, plasma levels of methionine, tryptophan and tyrosine are lowest in vegans, suggesting dietary deficiency for these AA. In addition, owing to their different structure and/or content in phytic acid and protease inhibitors, plant proteins are less digestible than animal proteins (17). Therefore, it has been suggested to consume different plant proteins with complementary essential AA composition to optimize a full pattern of dietary AA, especially for elderly subjects (17). In long-term studies, at low (≈0.8 g/kg/day) protein intake, plant proteins have lower ability to stimulate MPS and to cause muscle anabolism vs. animal proteins. However, the anabolic effect of plant- and animal-based proteins is similar if the protein intake is raised to 1.1–1.2 g/kg (17).

### Timing of Protein Ingestion

Protein consumption in western countries exceeds the Recommended Dietary Allowances (RDA), i.e., the protein amount that is be consumed daily to meet population needs and to prevent protein deficiencies. However, in many countries the intake is shifted toward the evening meal. In healthy subjects, the rate of MPS is 25% increased when food is distributed during the day, compared to a pattern with protein consumed at the evening meal (27). This finding may have important implications for increasing MPS in subjects who experience a resistance to the stimuli of MPS. Further, immediate post-exercise nutrient ingestion has been shown to increase the accretion of whole body and leg protein more than in a late (3-h) period (28).

Protein ingestion before sleep, if combined with physical activity, is also potentially anabolic (29) and has been suggested to represent a nutritional strategy to increase muscle mass in the elderly. However, there is no study in dialysis-treated patients.

## Physical Activity

In healthy subjects, resistance exercise augments the MPS rates, resulting in muscle hypertrophy (30) and also increases the expression of contractile, metabolic, and stress response proteins (31). In addition, muscle hypertrophy is associated with improved gait velocity and stair-climbing power even in very old individuals (32, 33). Aerobic fitness increases both markers of mitochondrial biogenesis and MPS (p-P70S6k) (34). In addition aerobic exercise training increases vagal tone and decreases sympathetic tone (35).

Early studies have shown that a single session of exercise increases MPS rates (36, 37). Despite this increase in MPS, if there is no food ingestion, the net muscle protein balance persists to be negative, even if to a lesser extent than in the pre-exercise period (37). During post-exercise recovery period, an increase in AA levels and supply to skeletal muscle from orally administered AA stimulates MPS and, even if to a less extent, inhibits MPD rates, resulting in muscle protein anabolism (21). Also the ingestion of carbohydrate improves muscle net protein balance (i.e., the difference between MPS and MPD) after resistance exercise; however, this effect is smaller as compared to protein/AA feeding (38). Therefore, post-exercise protein/AA ingestion is more commonly used to maximize anabolism. In addition to protein intake, body composition, age, and/or sex, and usual physical activity may influence the MPS response to feeding (28).

## Aging and Common Kidney Disease Comorbidities Which Impair MPS

Several conditions are recognized to impair the MPS response to protein, thus causing “anabolic resistance” and limiting the muscle hypertrophic response.

### Aging

The progressive aging-associated muscle atrophy, which frequently presents with muscle weakness and slow motion, is the most common type of muscle atrophy observed in humans (39). With aging, the risk of both CKD and declining capacities such as reduced strength and cognition increases (40); this risk is maximized in subjects developing “accelerated biological aging” (41). Sensory–motor functioning is very common in frail individuals who are at highly vulnerable for loss of independence in activities of day living, fall and mortality (42). Since the population in the western countries aged > 65 is expected to rapidly increase in the next years (43), CKD and age-related losses in skeletal muscle function and mass are considered as an extremely important public health issue (44).

Both quantitative and qualitative modifications of muscle biology and function are observed in elderly subjects. Early computed tomography studies (45) have shown that aging is associated with a loss of lean mass, with fat and connective tissues replacing muscle. These changes in muscle composition are considered to be consequence of a decreased MPS rates with aging (46).

The age-related muscle atrophy has been described as a slow but unrelenting process, with functional loss varying largely among individuals, which is observed in all elderly subjects, including those healthy and physically active (41, 47). Although the study of sarcopenia is complicated by muscle-specific differences among species and in the response to aging (41), there is consensus that anabolic stimuli do not efficiently activate the IGFI/PI3K/Akt/PKB/mTORC1 pathway (displayed in Figure 1) in elderly people, and this anabolic resistance is believed to play a major role in causing sarcopenia and reduced muscle recovery after an injury (41). In accordance with this view, acute activation of the pAkt/FoxO axis has been shown to be of benefit in the elderly (48). In addition, similarly to cancer cachexia, the age-associated low-grade systemic inflammation plays an important role in the pathogenesis of sarcopenia (49). How renal disease abnormalities overlap or potentiate those occurring with aging is still unexplored.

### Inactivity

Immobilization or sedentarism induces a status of “anabolic resistance” with reduced fasted and fed-state MPS. The effects of reduced use on skeletal muscle are in part similar to those observed during aging (50), and the increased sedentary behavior occurring in old age (51) has been suggested the be major cause of the aging-related sarcopenia.

Long-term immobilization in bed causes a more marked decrease in muscle strength than in muscle mass (52). However, changes at cellular and subcellular levels have shown that long-term immobilization causes a significant loss of contractile proteins, that is even greater than the decrease in muscle fiber force generation capacity (53).

### Low Energy Intake

A crucial aspect of the nutritional balance is the amount of energy ingested. From studies based on nitrogen (N) balance in the last century, it is known that the protein metabolism is influenced by the amount of energy intake (54–57). Of note, if dietary energy is supplied in amounts sufficient to meet body needs, protein intake strongly influences N balance. In healthy subjects, the administration of excess energy spares the loss of labile proteins, and reduces the time to adapt to a low protein diet. The supply of excess energy results in increased availability of ingested protein, with decrease in protein requirement (58). In keeping with this finding, N balance appears to be more influenced by changes in dietary energy supply at low protein intakes, and the N-sparing effect of energy is blunted when protein intake increases (59). These observations have practical implications, since protein requirements are determined when the requirements for energy are met.

To explain the mechanisms underlying the responses of protein metabolism to changes in protein intake, several years ago Young et al. (60, 61) studied whole body protein metabolism in 12 young men fed a low protein diet (0.6 g egg protein/kg/day) associated with a maintenance energy level or a 25% energy excess. In the fed-state the rate of leucine oxidation tended to be lower, while N balance and net protein balance were higher, when dietary energy was increased. These studies indicate that the oxidation of essential AA is sensitive and varies according to the levels of energy intake, with higher oxidation at low intakes.

HD patients have reduced dietary energy intake (62) and progressively lose muscle mass with time, even if body fat seems to be better preserved than lean mass (63). Studies on the dietary energy requirements of adult HD patients have been assessed by measuring energy expenditure by indirect calorimetry and/or by the use of N balance (63, 64). The results of these studies are not uniform. Some studies reported that dietary energy needs are not different from those of normal adults (65), whereas other studies showed increased energy expenditures (66). Inflammation has been reported to be associated with an increase in energy requirements (67). A few studies in HD patients have evaluated energy expenditure during both at rest and activities daily living. Shah et al. (68) measured dietary energy requirements for 92 days in 13 HD patients residing in a metabolic research ward while receiving a constant energy intake. Their average energy intake was 31 kcal/kg/day calculated from energy intake and change in fat and fat-free calories, which was 28 kcal/day. However, a wide variability in dietary intake among individual patients was observed (range: 26–36 kcal/Kg/day) (68). Dietary intake correlated strongly with their body weight, but was less closely related to their measured resting energy expenditure (REE). Overall, the data from this study show that, on the average, dietary energy requirements of sedentary, clinically stable HD patients are similar to those of age-matched sedentary normal subjects. However, due to the high individual variability in energy requirements, careful individual monitoring of the nutritional status of HD-treated patients is necessary (68).

Despite the importance of energy intake on protein requirements in N balance-based studies, the MPS response to food has been largely focused on the effects of orally administered protein or AA. However, food is usually eaten as a “mixed” meal. One of the main reasons for focusing on isolated protein ingestion is that protein provides essential AA for protein synthesis, while carbohydrates and fats ingested alone are only mildly muscle-anabolic. Protein absorption is delayed when carbohydrate are co-ingested with proteins, without however reducing the MPS response (69). However, protein and carbohydrate ingestion increase systemic insulin, which has a modest stimulatory effect on MPS and decreases MPD (38).

A few studies have focused on the effects of fat co-ingestion on the MPS response. Following physical exercise, the leg removes a greater amount of AA after the ingestion of high-fat milk compared with skim milk (70). In addition, the ingestion of whole eggs (18 g protein, 17 g fat) has been shown to cause a greater increase in post-exercise MPS rates than an isonitrogenous amount of egg whites or egg whites (18 g protein, 0 g fat) (71). This differential response may be due to the greater content of fat and/or micronutrients in whole eggs. Accordingly, fat co-ingestion (237 g of whole milk as compared to far-free milk) may increase the MPS response to protein in the post-exercise period (71). However, MPS is blunted by a high fat content (72, 73).

MPS is a process that “*per se*” requires energy (74). HD patients display muscle mitochondrial morphological and dysfunctional changes, which might limit the availability of energy for MPS or impair the efficiency of MPS in terms of energy costs. The energy requirements of whole-body PS have been estimated to account for ~20% of resting energy expenditure (REE) in humans (75). Since muscle protein turnover accounts for ~40% of whole-body protein turnover in humans, one could estimate the cost of MPS to be ~8–10% of REE. The cost of MPS is likely similar to control subjects in wasted HD patients. When both MPS and REE have been concurrently studied in non-inflamed, wasted HD patients before and during Growth Hormone (GH) administration (76) the variations in REE and MPS were significantly correlated suggesting that changes in MPS account for a significant fraction of REE changes. On that ground the energy cost of MPS was estimated to be ~5 kcal/g, which is similar to the normal condition (76).

### Diabetes Mellitus

Insulin is an anabolic hormone. Major effects of insulin's action are to suppress protein degradation and up-regulate anabolic pathways (77). While in type 1 diabetic subjects muscle wasting results from overexpression of genes involved in the ubiquitin proteasome pathway, in type 2 diabetes a decrease in insulin sensitivity favors muscle atrophy. A decrease insulin's response in muscle may take place owing to alterations in the insulin signaling pathways secondary to inflammation, glucotoxicity, increased circulating free fatty acids and metabolic acidosis (78, 79). Furthermore, the activation of new anti-anabolic or catabolic pathways, such as the myostatin/activin A system, may occur (79).

Patients with type 2 diabetic kidney disease often show a series of metabolic and nutritional changes linked both to diabetes and kidney failure, including insulin resistance and cardiovascular comorbidities (80), protein-energy wasting and sarcopenia (81). These patients undergo an increase in MPD, an effect primarily mediated by the ubiquitin-proteasome pathway (82, 83). On a kinetic basis, an increase in MPD is often associated with an increase in MPS. However, this seems not to be the case for diabetic kidney disease. In a recent study Zanetti et al. (84) observed that patients with diabetic kidney disease have a catabolic pattern of whole body protein turnover, associated with a 10% decrease in MPS (84). Therefore, multiple changes in protein metabolism, including an increase in protein degradation not associated with an adequately-matched change in protein synthesis account for accelerated wasting in patients with type 2 diabetic nephropathy.

### Sepsis

Chronic uremia has for long been recognized as a state of acquired immunodeficiency (85, 86). Sepsis is recognized as the second leading cause of death in ESRD (87, 88). Besides infections from indwelling catheters, numerous other factors, including aging, diabetes, hypoalbuminemia, immunosuppressive therapy, the dialysis procedure, uremia and increased leptin levels (89, 90) can favor infections in ESRD patients.

Sepsis causes a rapid and significant loss of body protein, in part due to development of an impairment of MPS alone or in association with increases in MPD (91). Septic cachexia has been defined as a life-threatening condition of metabolic inflammatory complex associated with multiple organ dysfunction (91). The metabolic changes induced by the systemic inflammatory response, including mitochondrial dysfunction and metabolic shift (91, 92), are closely connected by a wide array of signaling molecules. In several tissues, including skeletal muscle, the expression of pro-inflammatory cytokines is regulated by Toll-like receptors (TLRs) (92). During sepsis, muscle TLRs monitor for the presence of endotoxin (92, 93) and, when activated by microbial products, induce a local inflammatory response leading to the activation of pro-inflammatory genes. In addition, TLRs can be activated by heat shock proteins and/or endogenous signals of tissue injury (93). Recent observations suggest that TLR4 link the innate immunity activation with the uremic state in skeletal muscle (9). In predialysis CKD patients, an upregulation of TLR4 in muscle is predicted by the residual renal function, suggesting that endotoxins or danger-associated molecular patterns (DAMPS) produced or retained in the pre-uremic state mediate TLR4 activation (94). These findings suggest that in uremic patients muscle inflammation is due to an overexpression of TLR4 (94). The potential for septic cachexia to serve as a novel target disease state to improve clinical outcomes is reviewed elsewhere (92).

### Metabolic Acidosis

A normal bicarbonate serum concentration (serum HCO3^−^ between 24 and 26 mmol/l) is the recommended target in renal patients. While metabolic acidosis is one of the most important, yet treatable, among factors contributing to accelerated protein catabolism in dialysis patients (79), its effects on MPS are less established. In rats made acidotic (mean pH 7.22) by intragastric administration of NH_4_Cl (95), protein synthesis substantially decreased in muscle (96). In another study, induction of metabolic acidosis with NH_4_Cl in normal individuals (mean attained HCO3^=^ 16 mmol/l) caused a significant reduction in MPS, while albumin synthesis remained unchanged (95); conversely, chronic NH_4_Cl -induced metabolic acidosis (mean HCO3^=^ 15 mmol/l) was reported to significantly decrease the albumin fractional synthesis, without effects on MPS (97). Lofberg et al. (98) studied the effects of the correction of metabolic acidosis on muscle protein metabolism in 16 HD patients who were randomized to increase or decrease bicarbonate supplementation (blood bicarbonate levels increased from 17.8 to 27.1 mmol/l in the first, and decreased from 26.6 to 18.6 mmol/l in the second group). Muscle protein net balance improved when acidosis was corrected, mainly as a consequence of changes in protein degradation while protein synthesis was unaffected.

In consideration of these discordant findings, it is unclear if mild acidosis may affects MPS. Clearly, metabolic acidosis is potentially anti-anabolic, since it can decrease the release of GH and IGF-1 and induce insulin resistance (79). However, the effects of acidosis on the anabolic effects of protein feeding in dialysis patients have not been studied so far.

### Hemodialysis Procedure

The mechanisms underlying muscle wasting in HD patients involve a large number of factors, including inflammation, anorexia, metabolic acidosis, anabolic hormone resistance, and the loss of AAs and protein (mainly albumin) in the dialysate (99–104). HD acutely decreases MPS and upregulates protein degradation (99–101). Different mechanisms underlie this catabolic response, including losses of AAs and proteins into the dialysis fluid and the upregulation of protein catabolism by the interaction of immunocompetent blood cells with the dialysis membrane. Loss of AAs is very variable (from 4 to 12 g) (102–105), and is directly proportional to the AA plasma levels, dyalisate area and blood flow. In addition, protein losses into dialysate (2–3 g) as well as protein adsorption to the dialysis membrane and tubing can occur. Newer dialyzers with medium cut-off membranes, which are designed to improve clearance of middle molecules, are associated with increased albumin losses (106), even if with large variability (107).

A current hypothesis is that the inflammatory state observed in a significant number of HD patients is boosted by the HD procedure, which might increase the risk of cardiovascular complications and cachexia. Boivin et al. (108) showed that HD increases interleukin-6 (IL-6) and the caspase-3-mediated cleavage of actomyosin, the contractile protein made by the actin-myosin complex In addition, protein degradation was higher in patients during the HD procedure as compared the pre-HD period.

The activation of the complement with its downward inflammatory responses may be an additional mechanism leading to wasting in HD patients (109, 110). By a proteomic approach, Mares et al. (110), by eluting proteins adsorbed to the polysulfone dialyzer membranes, studied the processes that take place on the dialysis membranes. These proteins included ficolin-2 (a component of innate immunity that contributes to the immune recognition of pathogens), and complement C3c fragment. Of note, their data suggest that the polysulfone dialyzer initiates the lectin pathway of complement activation (111). In addition, a current hypothesis is that the anaphylatoxins C3a and C5a, bradykinin, thrombin and factor Xa can induce the activation of endothelial cells in the vascular wall, a process which may lead to inflammation and accelerated atherogenesis (112). However, there is a lack of controlled studies exploring the muscle proteolytic response to different filter materials.

### Peritoneal Dialysis

One of the untoward effects of PD is the loss of proteins and AAs into the dialysate effluent. Patients undergoing PD lose both AAs (1–3.5 g/day), and a consistent amount of proteins (5–10 g per day), mainly albumin (113–115). The continuous loss of proteins and AAs is estimated to account for almost one-third of the increase in dietary protein requirements, but poor appetite or anorexia limit intakes in many patients (116, 117). When muscle AA kinetics has been studied during the PD procedure, it was observed that PD causes an acute decrease in MPS, which has been associated to the combined effect of hyperinsulinemia and AA losses (118).

CKD has been considered for years a state of “anabolic resistance” (119). However, only a few recent studies give support to this hypothesis and show that the muscle anabolic response to protein ingestion is blunted in dialysis-treated patients.

CKD causes a series of gastrointestinal abnormalities. including motility disorders, gastric achlorhydria, pancreopathy and small bowel bacterial overgrowth, which may impair protein absorption as demonstrated by the use of a ^13^C protein breath test (120). At muscle level, Chen et al. observed that leucine-stimulated mTOR signaling (121) and GH-stimulated insulin-like growth factor-1 (IGF-I) expression (122) are partly attenuated in chronically uremic rats. In addition, the same authors observed that acute metabolic acidosis blunts the leucine-induced increase in MPS in rat muscle (123).

In patients with CKD, after protein ingestion the plasma AA paten to peripheral tissues is abnormal, with a more marked rise in non-essential AAs, as compared to essential AAs (124–126). Studies performed by whole body leucine kinetics in CKD patients have shown that whole body protein synthesis is resistant to stimulation by hyperaminoacidemia while the response of protein turnover to high insulin levels is normal (127). In addition, in patients with CKD and mild metabolic acidosis insulin sensitivity of muscle protein metabolism is overall preserved at insulin levels in the high (~8–9-fold increased vs basal) physiological range; however, muscle insulin's sensitivity in the low physiological range is impaired (128). This observation suggests that CKD patients may need higher insulin levels to decrease MPD after a low glycemic index meal, such as breakfast. In contrast, the muscle insulin's sensitivity at raised insulin levels, as may take place with larger meals containing refined carbohydrates, appears to be normally maintained.

Recently, van Vliet et al. (129) observed that in HD patients, the ingestion of a mixed-meal containing 20 g protein failed to increase MPS. Unfortunately, basal rates of MPS were ~2-fold increased in HD patients as compared to control subjects, a finding that might have impaired the ability of researchers to demonstrate any meal-induced MPS effect. In a recent study, Draicchio et al. (130) observed that whole body protein turnover rate decreased in HD patients after the ingestion of a mixed-meal compared with controls, suggesting that AA were poorly absorbed from the meal or dyregulated AA metabolism.

HD patients may also display “anabolic resistantance” to physical exercise. In the “PEAK” study Cheema et al. (131) randomized 49 patients to a 12-week resistance training during routine HD treatment or usual care. Physical exercise improved muscle strength, mid-thigh and mid-arm circumference, as compared to controls, but was not followed by statistically significant difference in muscle cross sectional area (an index of muscle hypertrophy). Mulsted et al. (132) studied 29 HD patients before and after completing 16 weeks of strength training. Before the training period, the participants were randomly assigned to receive a protein or a non-protein drink after every training session. Strength training did not result in muscle hypertrophy, while it was associated with increases in muscle strength and power (132).

Taken together, the available data in experimental models and in humans suggest that HD patients are resistant to the most important anabolic stimuli, i.e., protein and physical exercise. If so, HD patients, in addition to well-tailored exercise programs, need to eat more than the 20 g high-quality protein which is necessary to obtain an optimal MPS response in healthy subjects (8). As an example, elderly subjects, who are also considered to be “anabolic resistant”, need 35 grams of protein-i.e. ~18 g essential AAs) for the maximal stimulation of MPS in exercised and rested muscle (132, 133). However, this large amount of AAs, if not efficiently used for MPS, could boost ureagenesis, toxins and acid production. As an alternative, the use of keto acids (KA) of essential AA might offer an anabolic stimules with less N. It appears therefore urgent to obtain informations on the optimal dose of protein and/or oral AA/KA to maximize MPS in HD patients.

## Discussion: How to Overcome Anabolic Resistance in Dialysis-Treated Patients?

In this narrative review, we have briefly summarized our current evidence on factors which regulate MPS in humans, as a background information for effective treatment of patients with advanced CKD, mainly those on maintenance HD.

### Consider Amount, Quality, and Timing of Ingested Proteins

According to the current NKF KDOQI/AND guidelines, “nutritional counseling should aim to achieve a dietary intake of 1.2 g protein and 30–35 kcal/kg body weight/day in dialysis patients at risk of malnutrition” (134). This can be obtained through increased intake of nutrients, food fortification, and/or the provision of oral nutritional supplementation. However, despite strong attention to nutritional requirements, many dialysis-treated patients do not meet the current guidelines (1, 103, 104).

Quality of dietary proteins needs also to be checked. Plant proteins contribute to a large part of daily protein intake in the world population (135) and plant-based low protein diets are encountering increasing success for the prevention and treatment of several comorbidities associated with CKD (136). In addition, plant protein-based diets may correct many of the uremia-associated abnormalities (137–140). However, both in young and elderly subjects, plant proteins have a lower ability to increase MPS than animal proteins, when protein intake is low (≈0.8 g/kg/day) (17, 141). Of note, in elderly subjects, plant- and animal-based proteins are similarly anabolic when plant protein intake is adequate (1.1–1.2 g/kg) (17, 141), although no study is available in HD patients. Therefore, increasing the intake of plant protein to 1.2 g/kg may be the first option to offer when dietary plant-based protein content is low. Other approaches have been suggested to increase the anabolism induced by plant protein (141). These may include: 1) considering different plant protein sources; 2) combining animal and plant proteins; 3) supplementing plant-based diets with essential AA or KA of essential AA (141).

One of the current debates on nutritional treatment of dialysis patients is how to increase dietary protein intake without causing hyperphosphatemia, which is associated with all-cause and cardiovascular mortality (142). However, HD patients may have both poor nutritional status and hyperphosphatemia. Often nephrologists handle with hyperphosphatemia by prescribing both phosphate binders and restriction of protein-rich foods, an effect which may, however, boost wasting. Recently, Yamamoto et al. (143) observed that the practice of recommending an increase in dietary protein intake for HD patients with concurrent low serum albumin and high phosphate levels was associated with higher serum creatinine and potentially lower all-cause mortality in the Dialysis Outcomes and Practice Patterns Study (DOPPS) study-phase 4 (2009–2011, follow-up 1.6 years). According to this observation recommending the liberalization of protein intake together with hyperphosphatemia management may be a critical practice for better nutritional status and outcomes in HD patients. However, prospective RCTs are needed to determine the impact of protein intake liberalization on protein status and hard outcomes in malnourished, hyperphosphatemic HD patients.

### Allow Eating During HD

In many dialysis centers HD patients are fasting throughout the entire dialysis session, for fear of hypotension or vomiting (144). In addition, patients are often suggested not eating before the session, so it is very common that those on an afternoon HD session miss their noon meal (or they miss their breakfast if they have a planned morning HD session). For these reasons, nutrient intakes are often lower than recommended on dialysis-treatment days as compared with non-dialysis days.

In acute studies, feeding protein during HD treatment has been shown to cause a positive whole body protein balance (145). Providing meals during the HD treatment can increase overall nutrient intake and as well as the percentage of patients who reach nutritional targets (146). To replete AA pools, protein-rich foods have been recommended during and after the HD session; additionally, it is worth recognizing that wanting of protein-rich foods increases immediately after dialysis (147). Allowing patients to eat regularly during HD has been associated with better essential AA kinetics (148), nutritional status, quality of life, and possibly clinical outcomes [see for review ref. (149)].

In patients with gastrointestinal symptoms or who do not tolerate eating during HD treatment, intradialytic parenteral nutrition (IDPN) is used to prevent HD-related catabolism (150, 151), but the nutritional effects of this treatment may require long-time. Although a prospective study did not show an advantage of adding IDPN to oral nutritional supplementation on in malnourished HD patients, the improvement in serum prealbumin was associated with a decrease in morbidity and mortality (152).

Deleaval et al. (153) recently showed that the addition of branched-chain AAs (BCAAs) in the dialysis fluid may help to maintain their physiological plasma levels and MPS. Clearly, the clinical usefulness of this approach needs to be tested.

### Intraperitoneal AA + Glucose in PD Patients

PD shifts muscle protein metabolism to a status of reduced anabolism, a condition potentially harmful if nutrient intake is diminished or during superimposed diseases (154). Results from clinical trials on the use of intraperitoneal AAs (IPAA), including both RCTs and non-RCTs, were inconclusive [see ref. (121) for review] because of insufficient sample size and/or the occurrence of confounding conditions (121). Currently, IPAA are recommended when protein and energy intake are unattainable (121).

In an acute study (118), MPS was stimulated when AAs were supplemented intraperitoneally in combination with dextrose. The combined use of dextrose and AAs resulted in a cumulative anabolic and anticatabolic effect, because of the decrease in MPD (induced by insulin) and the stimulation of MPS (induced by AA availability) as compared to dextrose. Canepa et al. (155) in a study using an automatic PD cycler (APD) to simultaneously infuse glucose and AA (proportion of 3:1) in pediatric patients observed that the combined treatment was associated with a better metabolic profile, with maintenance of adequate non-protein calorie/nitrogen ratio. Therefore, these findings support the use of combined glucose-AA solutions, to improve utilization of AA for MPS while controlling urea levels (155). More recently, Tjiong et al. (156, 157) in an open label, cross-over study, compared the effects of a mixture of AA and glucose vs. glucose only containing dialysate, in 2 periods of 7 days each in eight APD-treated patients. They concluded that APD with dialysate composed of a mixture of AA and glucose acutely improves protein metabolism, and that this gain made during the night persists to a considerable extent for 24 h. However, no long term RCT is available on the effects of combined use of IPAA + glucose on nutritional status and harder outcomes.

### Oral Nutrition Supplements

The administration of oral nutritional supplements (ONS) is used to maintain nutritional status (156), when dietary counseling alone is not sufficient to fill the gap between actual intakes and target requirements. Patients are advised to take ONS preferably 1 h after meals rather than as a meal replacement (156). Essential AA supplements offer the possibility to increase MPS; many observational studies have shown that in the short term, ONS can improve albumin and blood EAA profile in HD patients (156). A recent meta-analysis in HD patients shows that if short-term (3 months) ONS use increases BMI and serum albumin levels, prolonged (**>** 3 months) supplementation does not causes such effects (158). Recently, Pokkrong et al. (159) observed that ONS administered to 80 HD patients was associated with an improvement in energy, protein, fat, fiber and magnesium intake and 29–24% decrease in malnutrition-inflammation score (MIS), while the improvement in serum albumin was slight (5.3–3.3%). A Cochrane meta-analysis (160) included 22 studies (1,278 participants, 79% on HD and 21% on PD); however, the authors pointed out that many studies were at unclear risk of selection, performance, and reporting bias. Overall, it is likely that protein-based ONS increase serum albumin and prealbumin (mainly in malnourished patients), as well as anthropometric measures. However, it is unclear if these results may translate into clinically relevant outcomes. In a recent meta-analysis Liu et al. (161) pointed out that large well-designed RCTs in this population are required. There is also little information on the effects of different AA formulas prescription.

### Ketoacids and Hydroxyacid Analogs

Even less information on the anabolic effect for ONS containing AA and KA is available in the HD or PD setting. Currently, AA and KAs containing supplements are associated to low protein diets (LPDs) or very-low protein diets (VLPDs) in patients with CKD4-5 (162, 163); these supplements have phosphate-chelating properties, are likely anabolic, can substantially postpone time to renal replacement therapy and decrease uremic toxicity (162); very recently, they have also been shown to protect patients with diabetic CKD from CV mortality (164).

Ketoacids (KA) of essential AA in general and ketoisocaproate (Kic) in particular, have been shown to reduce muscle protein degradation. In particular, in experimental models of CKD, a LPD supplemented with KAs compared to an LPD alone was able to downregulate the activity of the ubiquitin-proteasome system and to protect skeletal muscle from atrophy and oxidative damage (165). KAs can also maintain the activity of the mitochondrial electron transport chain complex and increase mitochondrial respiration (166). In the perfused heart muscle, branched-chain α-ketoacids (BCKA) increase the phosphorylation of the translational repressor 4E-BP1 as well as multiple proteins in the MEK-ERK pathway, leading to an increase in PS (167).

The Branched-chain amino acid transaminase (BCAT) enzyme reaction is reversible and maintains the equilibrium between the BCAA and their respective KA. Skeletal muscle contributes by the largest fraction of leucine deamination, reamination, and oxidation in the body in humans (168, 169). During fasting leucine is preferentially deaminated to Kic in muscle (168, 169). However, because of the reversibility of the BCAT reaction, the administration of KA can increase intracellular their respective AA level, mostly important the concentration of leucine. In an earlier study, Escobar et al. (170) observed that the infusion of Kic in the neonatal pig, stimulated MPS similarly to infusion of leucine. Both treatments increased intracellular leucine concentrations within the range 2–4-fold, a level considered to be essential for mTORC1 activation. More, recently it has been shown that BCKA are preferentially reaminated and activate protein synthesis in the heart (167) an effect that on a long term, could favor heart hypertrophy in conditions associated with elevated BCAA levels, such as obesity.

Recently, Fuchs et al. (171) were able to show that feeding old male subjects with 6 g BCAA, 6 g BCKA and 30 g milk proteins equally increased MPS; however, following the ingestion of milk protein MPS rates persisted to be high, while the postprandial MPS increase was short-lived following the ingestion of both BCAA and BCKA. On the one hand these findings suggest that a complete essential AAs pattern needs to be provided to allow a prolonged postprandial increase in MPS; on the other hand, it leads to the speculation that KA are truly anabolic and may be considered to treat PEW in HD patients. However, Li et al. (172) in a small prospective, randomized, controlled, single-center study observed that the KA supplementation did not improve neither plasma AA nor body composition. The ability to use a low-volume/small-quantity BCKA supplement to efficiently stimulate MPS, while avoiding excess N intake, warrants additional trials to define formulas, dosing and examine long-term clinically relevant outcomes.

### Endurance and Resistance Training

Early studies in HD patients have suggested that physical exercise prevents cardiovascular diseases, by ameliorating cardiovascular risk factors as well as cardiac autonomic control and left ventricular systolic function (173–175). In the HD-associated catabolic outline, it is not surprising that great interest has been given to physical exercise as a means to improve muscle strength and function. Kopple et al. (176), to study potential mechanisms by which exercise training ameliroates exercise capacity, randomized 80 HD patients into endurance training, strength training, endurance plus strength training or no training. At the end of the intervention, there was no whole body neither regional lean and fat mass in any groups. However, the investigators were able to demonstrate several muscle transcriptional changes that would favor muscle anabolism, including increases in muscle IGF-I and decrease in myostatin (176).

Among the treatment options to prevent loss of muscle mass and function in ESRD patients, endurance or resistance exercise appears to be the most useful. Several cohort studies have investigated the effects of endurance exercise training on physical function, aerobic capacity and muscle strength in HD patients. Results from these trials have showed benefits in terms of physical function, cardiovascular disease (CVD) risk, and quality of life (QOL) [see for review refs. (177, 178)]. Also expert opinion reports (179, 180), position statements (181) and guidelines (182) have suggested that physical exercise needs to be considered as standard of care treatment in patients with CKD, including HD patients. These indications are similar to recommendations for the general population (183).

Either “intradialytic cycling,” with patients cycling on ergometers during the HD session and out-of-center (“interdialytic”) exercise appear to have similar results (184). However, some apparently discrepant results have also been reported (185). Two major RCTs have been recently published on the effect of physical exercise in HD patients. Koh et al. (174) randomized 70 HD patients to intradialytic or home-based exercise training or usual care for 6 months. In the intradialytic arm, patients underwent three training sessions per week on a cycle ergometer, while in the home-based exercise arm patients were provided with a walking program to achieve the same weekly physical activity; the primary outcome was assessed as a change in performance on the 6-min walk test. Unfortunately, the authors found no significant differences among groups in the 6-month study period (174). More recently, Manfredini et al. (186), in a large 6-month randomized multicenter trial in HD patients, showed that personalized walking exercise program at home was associated with significant benefits on physical function, including the 6-min walking test, five times sit-to-stand test, cognitive function score and quality of social interaction score (186). Importantly, patients with the highest adherence to the exercise protocol had the largest performance improvement, suggesting a dose-response effect from the exercise. More recently, Exel et al. (187) studied 107 HD patients randomly divided into two groups: stretching and resistance exercise. Intervention programs were performed for 8 weeks, three times a week. Resistance exercise caused an increase in muscle strength and distance walked, as compared to stretching (187).

It has been outlined (185) that the reasons for the apparently discrepant results from training studies are possibly related to the need of enrolling a large number of patients to detect significant results. Another problem is the low volume and intensity of the exercise prescription, which may have accounted for the relatively modest functional improvements (185). So a current hypothesis is that exercise interventions might fail to produce clinically significant improvements in HD patients, primarily because the volume and intensity of the exercise prescribed is insufficient (185).

In summary, several meta-analyses (174, 177, 178, 184, 188, 189) show a statistically significant improvement in physical function and quality of life after 3–6 months of endurance exercise training in HD patients. However, no conclusion can be reached for elderly HD patients, since a few studies are existing focusing on them (190). In addition, a major problem stems from the low percentage of HD patients who are able to perform physical exercise. ESRD patient are more commonly sedentary and tend to avoid exercise (191); additionally, the dialysis population is more often aged, frail, depressive and with many comorbid conditions which are major obstacles to exercising. Finally, often the HD personnel has lack of expertise or resource to start an exercise program (192).

### Combining Nutrition and Physical Exercise

The combination of physical exercise and nutrition provides the strongest muscle anabolic stimulus in humans. In the elderly, who are “anabolic resistant” combining exercise with protein supplementation provides an enhanced anabolic response (193). Several studies have addressed the issue of the efficacy of this combined treatment in HD patients [see ref. (192) for review]. Likewise, early studies in HD patients has showed that a single bout of either endurance or resistance exercise enhances the anabolic response to nutritional supplements (194). However, the results from long-term interventions have been less favorable (132, 195–197).

Many factors are likely to contribute to the modest advantages of exercise in HD patients, including limited exercise prescriptions and intradialytic training (192). A greater exercise dose or enhanced nutritional support may be needed to demonstrate the potentiated additive benefits of these treatments (192). Clearly, defining the role of exercise in CKD remains a top research priority.

### Other Treatments to Increase MPS and Muscle Mass

#### Beta-Hydroxy-Beta-Methyl Butyrate

HMB is a downstream metabolite of leucine. HMB is present at low concentrations in muscle, and its turnover is about 0.7% of that of leucine (198). Better than being used alone, HMB is often consumed with other AAs or as part of a multiple ONS. Initial studies on the use of HMB in healthy volunteers demonstrated a high anabolic effect (199). Despite some RCTs have not detected any positive effects on muscle mass, strength and function from HMB supplementation, HMB is still considered a nutritional compound that may possess the potential to attenuate the rate of muscle loss in conditions of anabolic resistance (198, 200), probably as a long-term treatment (201).

There is very little information on HMB metabolism in CKD. A large percentage of plasma HMB is excreted into urine; however, from the observation that leucine and Kic pools are reduced in CKD4-5 (94), one would imply that also HMB is reduced in muscle. We also have no information on the effects of HD on HMB plasma levels. Since HMB molecular weight is low (m.w.118) it is likely removed by HD. HMB is available in many ONS on the market, but whether if it can promote anabolism in dialysis-treated patients is not known. Fitschen et al. (202) observed no significant effect of HMB supplementation on body composition, bone density, strength, fall risk and quality of life in a double-blind, placebo-controlled, randomized trial in 35 HD patients. However, on analysis of plasma HMB concentrations, 5 of 16 patients (31%) in the HMB arm were found to be non-compliant at 3 or 6 months (202). Therefore, the role of HMB supplementation in patients who are on dialysis is still not completely explored.

#### Omega 3 (n-3) Fatty Acids

Omega 3 (n-3) Fatty Acids (n-3 FA) have been shown in both young and older adults to have favorable effects on muscle insulin sensitivity, inflammation and anabolism (203–205). McGlory et al. (206), by utilizing intravenous hyperinsulinemic and hyperaminoacidemic clamps have shown that a moderate dose (~4 g) of n-3 FA supplementation augments MPS rates both in healthy young and older adults (206).

Deger et al. (207) studied the effect of the administration of n-3 FA (2.9 g/d) over 12 weeks on muscle protein turnover in HD patients with systemic inflammation. N-3 FA supplementation was associated with decrease of forearm MPD but did neither influence MPS nor muscle net protein balance. In a recent meta-analysis Rondanelli et al. (208) studied the effect of n-3 FA and docosahexaenoic acid (DHA) supplementation on fat free mass and physical performance in patients with various chronic diseases. Daily n-3 FA + DHA supplementation (from 0.7 to 3.36 g) decreased the time of Time Up and Go (TUG) test, and the fat free mass had an improvement trend which was however not statistically significant. Overall, these data suggest that n-3 FA + DHA might have a positive effect on physical performance, and that their use may improve some sarcopenia component.

#### Testosterone

Hypogonadism is commonly observed in men with CKD, which occurs as an effect of both depressed hypothalamic-pituitary-gonadal axis functionality and androgen synthesis (209, 210). Hypogonadism may contribute to several common adult complications in males with CKD (209, 210). Testosterone's downward signal acts to offset some of the catabolic pathways which are activated by uremia. Testosterone promotes satellite cell recruitment and increases MPS through stimulation of androgen receptors and activation of the insulin-like growth factor-1 (IGF-1) pathway (211). In humans, the anabolic action of testosterone is mediated via an enhancement of the efficiency of MPS, i.e., the MPS rate relative to the availability of AA (211). Long-term testosterone users develop hypertrophy of both Type I and Type II muscle fibers (211).

Low testosterone levels are associated with reduced fat free mass and muscle strength both in CKD and in HD patients (209, 210). Furthermore, Chiang et al. (212) recently were able to show that low plasma testosterone is strictly related to declining physical function, frailty, and muscle wasting in HD patients, which suggests that the CKD-associated decrease in testosterone levels may contribute to the procatabolic environment. However, current guidelines do not recommend routinely prescribing testosterone to all elderly men with low testosterone concentrations, but suggest that testosterone therapy is offered on an individualized basis, after explicit discussion of its potential risks and benefits, to elderly men who have symptoms suggestive of testosterone deficiency and consistently low morning testosterone concentrations (211).

#### Growth Hormone

Growth Hormone (GH) is anabolic both through the IGF-I downward pathway and a direct effect on skeletal muscle (213). GH deficiency is associated with muscle atrophy and GH administration causes muscle hypertrophy (214). GH treatment has been reported to have beneficial effects in elderly subjects (215). Although GH and IGF-I replacement in GH deficient adults has proven anabolic, this is not an unequivocal finding, possibly due to differences in dosage, and time on treatment (216).

Short stature is commonly observed in children with CKD, even after renal transplantation. The CKD-related GH insensitivity is characterized by deficiency of functional IGF-1, and can be overcome by the administration of supraphysiological doses of recombinant human GH (rhGH); long-term rhGH treatment stimulates IGF1 synthesis, increases longitudinal growth and likely improves adult height (216).

RhGH has been licensed for the treatment of CKD-related growth failure in Europe, North America and many other high-income countries (217). In children, CKD is associated to significant muscle wasting (217). Concurrent rhGH therapy causes higher leg lean body mass Z-scores, compared to untreated-children (218). Similarly, small randomized trials of rhGH in malnourished adult dialysis patients showed benefits including weight gain, improved MPS (219) and performance (220, 221). The OPPORTUNITY™ Trial (222) examined whether rhGH reduces mortality in hypoalbuminemic HD patients. Secondary end points were effects on number of hospitalizations, cardiovascular events, lean body mass (LBM), serum proteins, exercise capacity, QoL and adverse events. Although the OPPORTUNITY™ Trial was terminated early owing to slow recruitment, treatment with rhGH, compared to placebo, improved certain factors associated with cardiovascular disease risk factors, without adverse outcomes.

#### Denosumab

In postmenopausal women, a major role in the development of osteoporosis is played by the increased activity of receptor activator of nuclear factor kappa-B ligand (RANKL) (223). The binding of RANKL to its cognate receptor RANK drives a series of events triggering differentiation, activity, and survival of osteoclasts. Denosumab is a monoclonal antibody which binds RANKL, thus interfering with osteoclast differentiation, activation and survival. Denosumab is currently approved for the treatment of osteoporosis in postmenopausal women at risk of fracture. In is interesting that in elderly subjects osteoporosis and sarcopenia have similar risk factors, underscoring muscle-bone interactions, which may result in wasting, falls, and fractures. The combined existence of osteoporosis and sarcopenia (osteosarcopenia) is an emerging definition, which is cause of a significant health burden (224). Kirk et al. (224) observed in a large cohort of community-dwelling older adults that risk factors associated with osteosarcopenia include older age, female gender, physical inactivity, low body mass index, higher fat mass and the coexistence of chronic diseases, including CKD. Osteosarcopenia is suggested to be caused by reduced mechanical loading, and altered crosstalk between muscle, bone, and fat cells. It is interesting that, in addition to nutritional treatment and physical exercise, Denosumab, may be offered to osteosarcopenic patients (225). By inhibiting RANKL, Denosumab decreases bone resorption, increases bone mineral density (BMD), and reduces new fractures in postmenopausal women with osteoporosis (225). In a non-randomized study of community-dwelling older adults denosumab treatment improved balance, fear of falling, and physical function (226), suggesting that denosumab may increase muscle strength and mass.

Studies have examined the denosumab-activated pathways in skeletal muscle. RANK is also expressed in skeletal muscle and can activate the NF-κB pathway, which mainly inhibits AKT/mTORC1 signal and myogenic differentiation, leading to muscle loss (227, 228). Mice overexpressing RANKL (HuRANKL-Tg+) undergo muscle atrophy, lower limb force and maximal speed (228). Interestingly, the administration of denosumab to HuRANKL-Tg+ mice increases limb force and muscle mass, increases muscle insulin sensitivity and downregulates myostatin gene expression (228). These findings suggest that RANKL decreases, while its inhibition improves, muscle strength and insulin sensitivity both in osteoporotic mice and humans. In dialysis patients on might expect a similar effect, although no study is available.

#### Vitamin D

Vitamin D deficiency is associated with muscle functional impairment and falls (229). Even if the vitamin D receptor (VDR) is expressed at low levels in skeletal muscle, the deletion of VDR in the myocyte decreases muscle size and strength (230). Vitamin D has in muscle both genomic and non-genomic effects, which regulate cellular differentiation and proliferation. The non-genomic effects include the regulation of membrane calcium channels (which suggest a role for vitamin D in muscle contraction) mitochondrial function, insulin signaling and muscle substrate metabolism. In addition, studies conducted in cell culture systems and animals suggest that both vitamin D and conjugated linoleic acids (CLAs) stimulate MPS. Although some studies have shown that supplementation with vitamin D in the general population has a positive effect on muscle function, including athletic performance, falls and strength (231, 232), not all results are univocal. In a recent trial in 32 sedentary, older adults both Vitamin D and/or CLA supplementation, did not have effects on MPS (233). Similarly, there is not enough evidence to understand the role of vitamin D on musculoskeletal outcomes in the CKD population (231). However, given that this lack of evidence does not necessarily indicate no effect on musculoskeletal health (231), vitamin D might be considered to improve muscle strength and physical performance in renal patients, especially those who have low 25(OH)D plasma levels (<20 ng/mL-50 nmol/L).

#### Other Micronutrients

There is some evidence that the MPS response to feeding may be augmented by the use of micronutrients, mainly those contained in the yolk (vitamin D, vitamin E, vitamin A, zinc, selenium, and cholesterol) (234, 235). However, if and to what extent micronutrients potentiate the anabolic effects of protein feeding or physical exercise in dialysis-treated patients still needs to be established.

#### Other Treatments

Several other targets to increase MPS, such as targeting pro-inflammatory cytokines, manipulation of transforming growth factor (TGF) family members, satellite cells and stimulation of mitochondrial biogenesis, which are currently under study, are reviewed elsewhere (3, 236).

**Figure 2:**
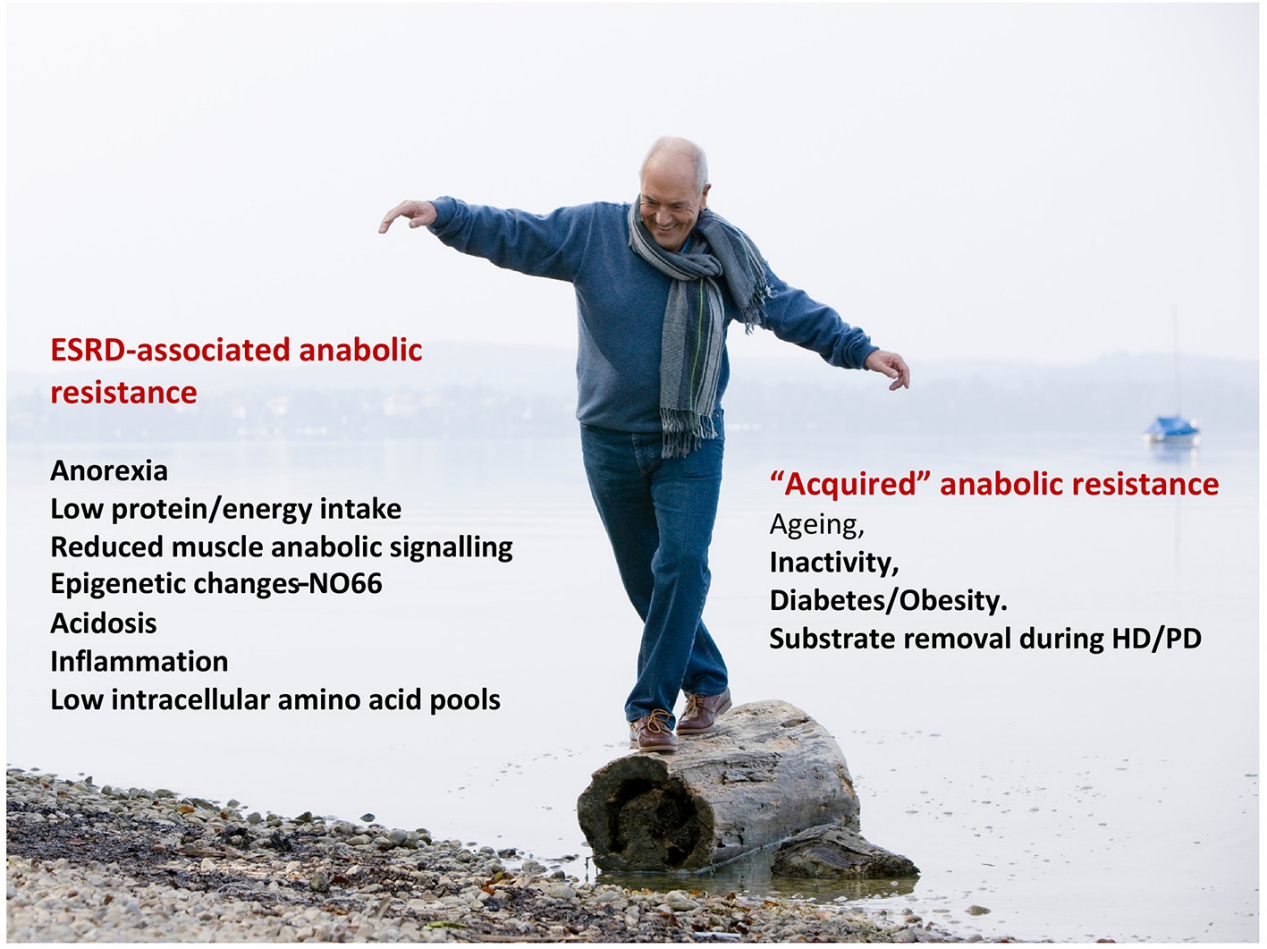
The dialysis patient balancing between the anabolic resistance associated to End Stage Renal Disease (ESRD) and the “acquired” anabolic resistance led by advancing age, inactivity, diabetes and substrate depletion during dialysis treatments (Shutterstock source).

In conclusion, the importance of maintaining muscle mass and MPS is often underlooked in patients with kidney diseases. HD patients are often exposed to AA or protein deprivation, which causes low circulating and tissue levels of essential AAs. Anorexia or fasting prescribed during the dialytic treatments can potentially decrease MPS. These settings associate with several abnormalities occurring in CKD that stimulate protein degradation and/or decrease MPS. Recent observations suggest that skeletal muscle is “anabolic resistant” in HD patients and that greater amounts of proteins than in healthy subjects are needed to maximally stimulate MPS. Accordingly, the dialysis patient needs to cope with the CKD-associated intrinsic anabolic resistance, and the “acquired” anabolic resistance led by advancing aging, inactivity, diabetes and substrate depletion (Figure 2), The combination of physical exercise and protein/AA feeding provides the strongest muscle anabolic stimulus in humans; however the major gaps in our current knowledge of nutritional treatment of dialysis-treated patients with CKD include optimal formulas and amount of protein, ONS and exercise paradigms, and research on how to incorporate effective management approaches into clinical care.

## Author Contributions

GG and DV conceived the review. MS and FA performed literature research. GG, FV, GB, and DV wrote the manuscript. ER, DP, AL, and PE reviewed and edited the manuscript. All authors have read and agreed to the published version of the manuscript.

## Conflict of Interest

The authors declare that the research was conducted in the absence of any commercial or financial relationships that could be construed as a potential conflict of interest.

## Publisher's Note

All claims expressed in this article are solely those of the authors and do not necessarily represent those of their affiliated organizations, or those of the publisher, the editors and the reviewers. Any product that may be evaluated in this article, or claim that may be made by its manufacturer, is not guaranteed or endorsed by the publisher.
